# Intracellular domain of epithelial cell adhesion molecule induces Wnt receptor transcription to promote colorectal cancer progression

**DOI:** 10.1186/s12929-024-01057-y

**Published:** 2024-07-15

**Authors:** Sushree Shankar Panda, Chi-Chiu Lee, Khamushavalli Geevimaan, Kai-Chi Chen, Shung-Haur Yang, Chia-Ning Shen, Wei-Chun HuangFu, Han-Chung Wu

**Affiliations:** 1 Institute of Cellular and Organismic Biology, Academia Sinica, No. 128, Academia Road, Section 2, Nankang, Taipei, 115 Taiwan; 2 Ph.D. Program for Cancer Molecular Biology and Drug Discovery, College of Medical Science and Technology, Taipei Medical University and Academia Sinica, Taipei, Taiwan; 3 Genomics Research Center, Academia Sinica, Taipei, 11529 Taiwan; 4 Division of Colon and Rectal Surgery, Department of Surgery, Taipei Veterans General Hospital, Taipei, 11217 Taiwan; 5 National Yang-Ming Chiao Tung University Hospital, Yilan, 260002 Taiwan; 6 School of Medicine, National Yang-Ming University, Taipei, 112304 Taiwan; 7 Biomedical Translation Research Center (BioTReC), Academia Sinica, Taipei, 11529 Taiwan

**Keywords:** EpCAM, EpICD, Wnt receptors, hEpAb2-6, Wnt signaling

## Abstract

**Background:**

Epithelial cell adhesion molecule (EpCAM) has been widely studied as a tumor antigen due to its expression in varieties of solid tumors. Moreover, the glycoprotein contributes to critical cancer-associated cellular functionalities via its extracellular (EpEX) and intracellular (EpICD) domains. In colorectal cancer (CRC), EpCAM has been implicated in the Wnt signaling pathway, as EpICD and β-Catenin are coordinately translocated to the nucleus. Once in the nucleus, EpICD transcriptionally regulates EpCAM target genes that; however, remains unclear whether Wnt signaling is modulated by EpICD activity.

**Methods:**

Patient-derived organoids (PDOs), patient-derived xenografts (PDXs), and various CRC cell lines were used to study the roles of EpCAM and EpICD in Wnt receptor expression. Fluorescence and confocal microscopy were used to analyze tumors isolated from PDX and other xenograft models as well as CRC cell lines. EpCAM signaling was intervened with our humanized form of EpCAM neutralizing antibody, hEpAb2-6. Wnt receptor promoters under luciferase reporters were constructed to examine the effects of EpICD. Luciferase reporter assays were performed to evaluate promoter, γ-secretase and Wnt activity. Functional assays including in vivo tumor formation, organoid formation, spheroid and colony formation experiments were performed to study Wnt related phenomena. The therapeutic potential of EpCAM suppression by hEpAb2-6 was evaluated in xenograft and orthotopic models of human CRC.

**Results:**

EpICD interacted with the promoters of Wnt receptors (FZD6 and LRP5/6) thus upregulated their transcriptional activity inducing Wnt signaling. Furthermore, activation of Wnt-pathway-associated kinases in the β-Catenin destruction complex (GSK3β and CK1) induced γ-secretase activity to augment EpICD shedding, establishing a positive-feedback loop. Our hEpAb2-6 antibody blocked EpICD-mediated upregulation of Wnt receptor expressions and conferred therapeutic benefits in both PDX and orthotopic models of human CRC.

**Conclusions:**

This study uncovers relevant functions of EpCAM where Wnt receptors are upregulated via the transcriptional co-factor activity of EpICD. The resultant enhancement of Wnt signaling induces γ-secretase activity further stimulating EpICD cleavage and its nuclear translocation. Our humanized anti-EpCAM antibody hEpAb2-6 blocks these mechanisms and may thereby provide therapeutic benefit in CRC.

**Supplementary Information:**

The online version contains supplementary material available at 10.1186/s12929-024-01057-y.

## Background

Epithelial cell adhesion molecule (EpCAM) is expressed in epithelial tissues and in various populations of tissue stem cells, precursors and embryonic stem cells. In addition, EpCAM is abundantly expressed thus has been studied extensively in many cancer types. The glycoprotein is activated by cleavage at the cellular membrane causing release of the extracellular (EpEX) and intracellular (EpICD) domains by a process called regulated intramembrane proteolysis (RIP). These cleaved domains are known to participate in a wide variety of cellular functions including some that promote cancer progression and metastasis [1–6]. Moreover, EpEX was reported to directly bind to EGFR and HGFR, stimulating receptor phosphorylation and activating the corresponding downstream signaling pathways [5–7]. It was further shown that the EpEX-EGFR-ERK1/2 signaling axis enhanced RIP of EpCAM in order to release EpICD promoting tumor progression [5]. At the same time, EpEX binding to EGFR activated AKT and MAPK signaling cascades, which respectively inhibited FOXO3a function and stabilized PD-L1 protein [8]. As such, the EpCAM-neutralizing antibody, EpAb2-6, was shown to inhibit AKT and FOXO3a phosphorylation, increase FOXO3a nuclear translocation, and upregulate HtrA2 expression to promote apoptosis, while also decreasing PD-L1 protein levels to enhance the cytotoxic activity of CD8^+^ T cells [8, 9]. Meanwhile, the interaction of EpEX with HGFR activates downstream signaling by inducing the FAK-AKT axis, thereby decreasing GSK3β activity to stabilize Snail protein and ultimately promoting metastasis [6]. Besides its involvement in such signaling pathways, EpCAM has also been shown to be a cancer stem cell (CSC) antigen that promotes tumorigenesis [2, 4].

One pathway known to play a central role in tumor progression and CSC function is Wnt/β-Catenin signaling. Wnt signaling is also crucial for many developmental processes and sustains adult tissue homeostasis, as it is an integral driver of cell proliferation, migration, differentiation and pluripotency of tissue stem cells. In line with these essential functions, dysregulation of Wnt signaling can result in a multitude of disease states including cancer and the pathway is thought to be a promising therapeutic target [10, 11]. With regard to its role as a central contributor to tumorigenesis, Wnt signaling has been shown to promote several key malignancy-associated characteristics, such as tumorigenic potential, tumor plasticity and drug resistance [11, 12]. Intriguingly, EpCAM has been reported as a mediator of canonical Wnt signaling, as EpICD acts at least partially with Wnt signaling components to promote cell motility, proliferation, survival and metastasis [1, 3–5, 13]. Canonical Wnt signaling is often activated by the interaction of external Wnt ligands with their cognate receptors, such as Frizzleds (FZDs) and LRP5/6. The ligand-bound receptors then recruit β-Catenin destruction complex to the cell membrane, causing release of active β-Catenin and allowing its translocation to the nucleus [11]. Soluble EpICD is known to form a multi-protein nuclear complex with active β-Catenin and a scaffolding protein called Four and one-half LIM domains protein 2 (FHL2). This assembled protein complex translocates to the nucleus, where it associates with T-Cell Factor (TCF) or Lymphoid Enhancer Factor 1 (LEF-1) and DNA to modulate transcription of EpCAM targets in a manner reminiscent of canonical Wnt signaling [1, 13–15]. In colorectal cancer (CRC) patients, high EpCAM expression suggests poor outcomes, in line with the known critical involvements of EpICD in CRC cell function [4, 8, 16–18]. Furthermore, EpCAM was previously shown to promote CRC cell invasion by EpICD-mediated activation of EMT-inducing genes, such as *Snail1*, *Slug* and *Twist*. In parallel, nuclear translocation of the EpICD-β-Catenin complex upregulates transcription of cell fate reprogramming genes, such as *Oct4*, *Sox2* and *c-Myc* that confer self-renewal ability to CRC cells [4, 19].

Since EpICD functions in a complex with β-Catenin, EpCAM activity is intimately associated with canonical Wnt signaling [1, 13–15, 19]. Furthermore, it is known that Wnt signaling components are abundantly expressed and aberrantly regulated in CRC. For instance, high levels of external Wnt ligands drive robust Wnt activity in CRC, and high expression levels of Wnt receptors (FZDs and LRP5/6) sustain Wnt signaling and further contribute to CRC cell propagation [11, 20]. Considering the association between Wnt signaling and EpICD nuclear translocation and binding to target gene promoters, we set out to explore whether EpICD might transcriptionally modulate Wnt pathway regulators and components, such as Wnt receptors.

## Materials and methods

### Patient derived organoid (PDO) culture

PDOs used in this study were taken from our pre-established live organoid bio-bank (administered by Prof. C.N. Shen), and the culture methods were performed as described previously [21]. Briefly, PDOs were embedded in matrigel and cultured in basal culture medium. The basal culture medium (1 × Advanced DMEM-F12, 1 × N2, 1 × B27, 1.25 mM N-Acetyl Cysteine, 1 × GlutaMax, 10 mM HEPES, 1% bovine serum albumin) was supplemented with 20% R-Spondin1 conditioned medium, 10% Noggin conditioned medium, 10 mM Nicotinamide, 50 ng/ml human EGF, 10 nM Gastrin, 500 nM A83-01, 3 M SB202190, 10 nM Prostaglandin E2, and 100 mg/ml Primocin (Invivogen).

### Organoid treatment, number and size measurement

PDOs were treated with either IgG or the humanized EpCAM-neutralizing antibody, hEpAb2-6 (20 μg/mL) (produced in-house), as indicated in the figure legends. Light microscopy (Olympus) was used to visualize the cultures, and images were captured with a camera attached to the microscope. The microscopy images were used to count organoid numbers and measure organoid size with ImageJ (FIJI) software.

### Cell culture

Experiments were performed using HCT116, HT29, CT26 and HeLa cell lines. HCT116, HT29 and HeLa cells were cultured in Dulbecco's Modified Eagle Medium (DMEM) (Gibco). CT26 cells were cultured in RPMI1640 (Gibco). Media were supplemented with 10% Fetal Bovine Serum (FBS, Gibco), 1% L-glutamine (Gibco), and 1% penicillin and streptomycin (P/S) (Gibco). All cells were grown in 5% CO_2_ at 37 °C. For the growth curve experiments, 10^4^ cells were seeded on 6-well plates in triplicate for each cell line. The cells in each well were counted using a hemocytometer, and the daily counts were averaged for days 1 to 8. After collection of the entire dataset, points were plotted to analyze the growth curve and calculate cell doubling time.

### Western blotting

For western blotting, PDOs/cells were lysed using radioimmunoprecipitation assay (RIPA) buffer (0.01 M sodium phosphate, pH 7.2, 150 mM NaCl, 2 mM EDTA, 50 mM NaF, 1% Nonidet P-40, 1% sodium deoxycholate, and 0.1% SDS) containing phosphatase inhibitor (Roche) and protease inhibitor (Roche) cocktail. Equal amounts of protein from each sample were separated by SDS-PAGE and then transferred to PVDF membranes. The membranes were blocked with 3% BSA in TBST (blocking solution) and incubated with appropriate primary antibodies in blocking solution overnight at 4 °C. Membranes were then incubated with HRP-conjugated secondary antibodies in the blocking solution for 1 h at room temperature, and protein expression levels were measured. Antibodies included anti-α-tubulin (Sigma), anti-EpCAM/EpICD (abcam), anti-Frizzled 6 (CST), anti-Frizzled 7 (Santa Cruz Biotech), anti-LRP5, anti-LRP6 (CST), anti-Presenilin2 (abcam) and anti-phospho-Presenilin2 (S327) (abcam).

### Wnt activity

Cells were plated at 5 × 10^3^ cells per well and grown overnight in 12-well plates. Then, cells were transiently transfected with TOP-Flash TCF reporter plasmid (Millipore) using poly-jet transfection reagent (SignaGen). At 48 h post-transfection, cells were treated with 20 μg/mL hEpAb2-6 for 6 h, or as indicated in the figure legends. Further, cells were lysed, and the luciferase assay was performed using a Luciferase assay kit (Promega).

### Immunohistochemical (IHC) staining

Human colon cancer tissue arrays were purchased from Biomax. Sections were de-waxed in xylene and rehydrated through a series of solutions with decreasing alcohol concentrations. Antigen retrieval was performed concomitantly in the Trilogy™ (Cell Marque) system. For peroxidase blocking, sections were incubated with methanol containing 3% H_2_O_2_ for 20 min at RT. Sections were further washed with PBS and incubated with 1% BSA in PBS for 30 min at RT to block non-specific binding. Subsequently, primary antibody [anti-active β-Catenin (Millipore) and anti-EpCAM antibody EpAb3-5 (produced in-house)] was applied, and samples were incubated at 4℃ overnight. Next, sections were washed with PBS containing 0.1% Tween 20 (PBST0.1) (Thermo) and treated with the Super Sensitive Super Enhancer reagent for 20 min at RT. Then, samples were rinsed three times with PBST0.1. Sections were subsequently treated with Polymer-HRP reagent for 30 min at RT and then rinsed three times with PBST0.1. Next, 3,3'-Diaminobenzidine (DAB) was used as a chromogen to visualize peroxidase activity. The quantification of protein intensities was performed using Fiji-ImageJ software.

### Immunofluorescence staining

Glass slides were placed in 24-well plates and coated with 0.1% gelatin. Then, 3 × 10^4^ cells were seeded in serum free medium overnight. Cells were treated as indicated in the figure legends. Cells were washed using ice-cold PBS and fixed with 4% paraformaldehyde for 15 min at RT, followed by washing with ice-cold PBS. Further, cells were permeabilized using 0.1% Triton-X in PBS for 20 min and subsequently washed with PBS. Cells were blocked with 3% BSA in PBS for 1 h at RT. Next, cells were treated with primary antibody [anti-Active β-Catenin (Millipore) or anti-EpICD 4A7 (abcam)] overnight. The samples were washed and treated with secondary antibody in PBS containing 3% BSA along with DAPI for 1 h at RT. Samples were further washed in PBS five times and mounted for microscopy. Nuclear β-Catenin or EpICD intensities were calculated using IMARIS (Oxford Instruments) software.

### Immunofluorescence (IFS) with tumors isolated from animals

#### Tumor isolation and paraffin embedment

Tumors, either from patient derived xenografts (PDX) or HCT116-xenografts, were surgically removed from the animals and fixed using 4% paraformaldehyde. Further, samples were dehydrated and embedded in paraffin. Sectioning of paraffin-embedded tumors was performed using a microtome (4 µM thickness), and the sections were fixed to glass slides.

#### IFS using tissue slides

Initial protocols were followed as described earlier in IHC section. After blocking with 1% BSA, sections were incubated with primary antibodies against EpICD (4A7, abcam) and against Wnt receptors [FZD6 (Bioss), LRP5 (abcam) and LRP6 (Santa Cruz)] overnight at 4 °C. Further, sections were washed with PBST0.1 and incubated with secondary antibody cocktail along with DAPI for 1 h at room temperature. Finally, the samples were washed with PBST0.1 and then mounted for microscopy. Quantification of protein intensities was performed using IMARIS software (Oxford instruments).

### Quantitative real time PCR (qPCR)

Total RNA was extracted using TRI reagent; 5 μg of the total RNA was then reverse-transcribed using oligo (dT) primer with reverse transcriptase. Quantitative real time RT-PCR (qPCR) was performed on cDNA using the Light Cycler 480 SYBR Green-I Master kit and the LightCycler480 System. The expression levels of each gene of interest were normalized to the expression levels of glyceraldehyde 3-phosphate dehydrogenase (GAPDH) or β-actin in the same sample. The primers used for qPCR are listed in Table S1.

### Tumor sphere assay

EpCAM knockout cells were transiently transfected with pcDNA or EpICD plasmid using polyjet transfection reagent (SignaGen). Cells were seeded in ultra-low attachment 6-well plates (5 × 10^4^ cells per well) or 24-well plates (1 × 10^3^ cells per well) (Corning) and maintained in DMEM/F-12 (Gibco) supplemented with B27. Cells were cultured for 10 days (medium changed every two days), and on the tenth day, spheres were counted and photographed under microscopy.

### Colony formation assay

After transfection, cells were seeded in 12-well plates (5 × 10^3^ cells per well). The medium was changed every 2 days, and the cells were allowed to grow for 10 days. On the tenth day, cells were washed and fixed with 4% paraformaldehyde and further stained with 1% crystal violet for 30 min. Colonies were washed three times with PBS, and images were captured. To measure colony density, the wells were incubated with shaking in 0.5% SDS for 2 h at RT. The supernatants were collected, and the absorbance of the solution was measured at 570 nm using a microplate reader.

### Apoptosis assay

Cells were seeded in 24-well plates (5 × 10^4^ cells per well) overnight and then treated with 20 μg/mL hEpAb2-6 or as indicated. Cell pellets were collected, and an apoptosis assay was performed using an Annexin-V/PI apoptosis kit (BD Biosciences). Signals were detected by flow cytometry analysis, and the percentage of apoptotic cells was calculated.

### Luciferase reporter assay

Cells were seeded in 24-well plates (1 × 10^4^ cells/well) and incubated at 37℃ for 24 h. After culture media were refreshed, the cells were transfected with reporter plasmids (γ-secretase, TCF reporter or Wnt receptor promoter reporter) using PolyJET (SignaGen). Similarly, PDOs were dissociated into single cells and then transiently transfected with indicated reporter plasmids before being seeded to form new organoids (see further details in figures and legends). In both PDOs and cell lines, the transfection efficiencies were controlled by normalizing to co-transfected pRL-TK (20 ng) as an internal control. Additional treatments were conducted as indicated. Firefly luciferase and Renilla luminescence were measured 48 h post-transfection using the Dual-Glo Luciferase Assay System (Promega), according to the manufacturer’s recommendations.

### Tumorigenic potential in vivo

NSG mice were divided into two groups with equal numbers. EpCAM-control or EpCAM-knockout cells were subcutaneously transplanted (10^5^ cells) in the right flank of each animal (*n* = 6 in each group). Tumors were allowed to grow, and tumor dimensions were measured twice a week using slide calipers. Once the tumor volume reached 2000 mm^3^ (as specified by IACUC, Academia Sinica) for any mouse in the experiment, all animals in the experiment were sacrificed, and tumor weights and volumes were measured. None of the data was excluded.

### γ-Secretase activity

γ-Secretase activity was measured using the protocol described by Liao et al. (2004) [22]. In brief, cells were transiently transfected with the control plasmid and tetracycline-inducible γ-secretase plasmid harboring luciferase (plasmids were generous gifts from Dr. Yung-Feng Liao, ICOB, Academia Sinica). Cells were then treated with EpEX-His (250 ng/mL) (produced in-house with an Expi293 Expression System) or Wnt3A (R&D Systems) (100 ng/mL) for 4 h. Similarly, PDOs were treated with 20 μg/mL hEpAb2-6 after organoid formation. PDOs and cells were lysed using passive lysis buffer and subjected to the luciferase assay.

### Wnt receptor promoter reporter plasmid construction

The putative promoter regions of LRP5 (-1187 to + 200), LRP6 (-1543 to + 55) and FZD6 (-1385 to + 205) were cloned from HeLa genomic DNA and inserted into the pGL4.18 plasmid (Promega, USA). The genomic DNA was extracted using a Genomic DNA Isolation Kit (NovelGene, TW) according to the manufacturer’s recommendations. The primers used to generate PCR fragments of Wnt receptor promoters are listed in Table S2.

### GSK3 and CK1 activity on presenilin2 (PS2) phosphorylation

To assess the levels of GSK3 and CK1 kinase activities, 10^6^ cells were seeded overnight and treated with GSK3 inhibitor BIO (Sigma) or CK1 inhibitor PF-670462 (selleckchem) for 8 h. Cells were then lysed with RIPA buffer and subjected to western blot analysis for phosphorylated Presenilin2. Otherwise, cells were treated with EpEX (produced in-house) or recombinant Wnt3A (R&D systems) for 8 h before measurement of phosphorylated ADAM17 and presenilin2 (PS2) levels. Similarly, PDOs were treated with hEpAb2-6 and further lysed with RIPA buffer. The samples were then subjected to western blot analysis for phosphorylated PS2.

### Tumor transplantation and therapeutic studies in mice

All animal experiments were approved and performed according to the regulations from the IACUC at Academia Sinica. Procedures were performed as follows.

#### PDX model generation and treatment

PDOs were gently disrupted with TrypLE Express and passed through a cell strainer to produce a single-cell suspension. Cells were then mixed with matrigel (1:1) and subcutaneously transplanted (5 × 10^5^ cells) in the right flank of male NOD/SCID mice (6–8 weeks old). Animals were randomly divided into two treatment groups (*n* = 5 for each treatment condition), and the PDX tumors were allowed to grow for 2 weeks (until the tumor volume reached 80–100 mm^3^). Animals were then treated for four weeks with 20 mg/kg IgG or hEpAb2-6 via tail vein injection twice a week. When the tumor volume reached 1500 mm^3^, the endpoint was considered to have been reached. The animals were sacrificed at this point and survival times (days) were recorded.

#### Orthotopic model generation and treatment

A total of 2 × 10^5^ HCT116 cells with luciferase overexpression were surgically transplanted into the cecum wall of the animals. Male NOD/SCID mice (6 to 8 weeks old) were used for the experiments (*n* = 5 for each treatment condition). At 72 h post-transplantation, mice were randomly distributed into two treatment groups. For the treatments, animals were injected with 20 mg/kg IgG or hEpAb2-6 via tail vein injection twice a week for four weeks. Tumor progression was monitored using bioluminescence imaging. To image the tumors, intraperitoneal injection of D-Luciferin (GOLD BIO) was performed, and images were taken 10 min post-injection.

### Statistics

Statistical analyses were performed using GraphPad Prism (GraphPad Software 10). Firstly, normality of datasets was tested using the Kolmogorov–Smirnov test. Data sets of two groups with normal distributions were analyzed using a two-tailed Student’s t-test; otherwise, a Mann–Whitney U test was performed for nonparametric data. Lognormal data of more than two groups were tested using one-way ANOVA followed by Tukey’s multiple comparison test. For non-parametric distributions, a Kruskal–Wallis followed by Dunn’s multiple comparison test was performed. When appropriate, a two-way mixed model ANOVA was performed as indicated in the figure legends. Data were represented as all data points with the median value indicated; *P* values below 0.05 were considered statistically significant.

## Results

### EpCAM is associated with Wnt receptor expression in CRC

**Fig. 1:**
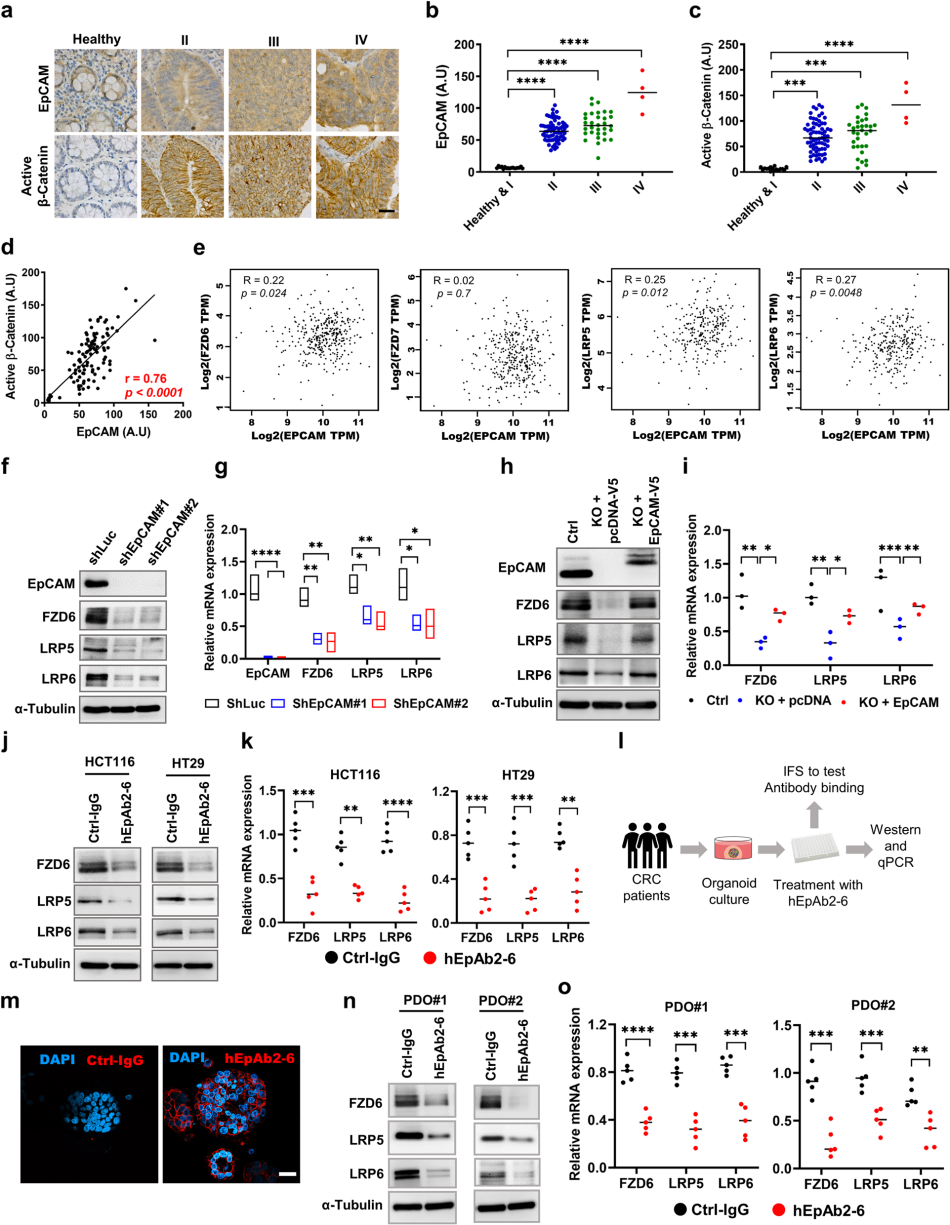
EpCAM is involved in Wnt receptor expression. **a** IHC staining for EpCAM and active β-Catenin in CRC of different stages. For each patient in the array, the same field of view is shown for both images. Scale bar: 20 μm. Quantification of expression intensities in samples for **b** EpCAM and **c** active β-Catenin expression. **d** Expression of EpCAM correlates with active β-Catenin in 120 patient samples (*n* = 120) corresponding to representative images in (**a**). **e** Correlation of mRNA levels for EpCAM and indicated Wnt receptors, computed from TCGA database. **d**, **e** show Pearson correlation coefficient r. **f** Western blot analysis showing indicated Wnt receptor protein expression levels and **g** relative mRNA expression levels in EpCAM knockdown H29 cells. **h** Protein levels and **i** mRNA expression levels in EpCAM-KO HCT116 cells transfected with either pcDNA or EpCAM-V5 plasmid. **j** Protein levels and **k** mRNA expression levels in indicated cell lines treated overnight with either ctrl-IgG or hEpAb2-6 (20 μg/mL). **l** Experimental design for use of patient derived organoids (PDOs). **m** IFS for hEpAb2-6 binding to EpCAM in PDOs (Scale bar: 30 μm). **n** Western blot analysis showing indicated Wnt receptor levels, and **o** corresponding relative mRNA expression levels in indicated PDOs. Quantified band intensities from three independent experiments (**f**, **h**, **j** and **n**) were provided in Supplementary Fig. 1. Data were analyzed using (**b**, **c**) Kruskal–Wallis test followed by Dunn’s and **g**, **i** one-way ANOVA followed by Tukey’s test for multiple comparison and **k**, **o** two-tailed t test. *p < 0.05, **p < 0.01, ***p < 0.001, ****p < 0.0001. Ctrl: control, KO: knockout

It is well established that CRC cells rely heavily on Wnt signaling activation for the disease progression. In canonical Wnt pathway, interactions of Wnt ligands with their receptors (at least one FZD and one LRP) recruit β-Catenin destruction complex to the cell membrane resulting de-phosphorylation of β-Catenin (activate form) [11]. Therefore, we began this study by asking if EpCAM expression was correlated with active β-Catenin levels in CRC patient tissue samples. To answer this question, we performed immunohistochemistry (IHC) on patient tissue samples and found that the levels of both EpCAM and active β-Catenin were elevated in disease samples compared to healthy tissues (Fig. 1a-c). A further correlation analysis showed that the expression of EpCAM was strongly associated with that of the active β-Catenin (Pearson’s correlation co-efficient *r* = 0.76, *p* < 0.0001) (Fig. 1d). Of note, Wnt receptor expression levels and other microenvironmental factors are known to influence Wnt signaling activity [20]. We thus analyzed datasets from the TCGA database to test whether a transcriptional correlation existed between EpCAM and commonly expressed Wnt receptors in CRC such as FZD6/7 and LRP5/6. The data revealed a significant positive correlation between EpCAM and Wnt receptors FZD6 and LRP5/6 but not between EpCAM and FZD7 (Fig. 1e). Therefore, we carried out further experiments to delineate FZD6 and LRP5/6 expression characteristics and regulatory mechanisms.

### EpCAM is involved in Wnt receptor protein expression

We first performed knockdown or knockout of EpCAM in established CRC cell lines and found that Wnt receptors were significantly decreased at protein and mRNA levels in the EpCAM-deficient cells (Fig. 1f-i and Supplementary Fig. S1a, b). Furthermore, transfecting knockout cells with wild-type EpCAM plasmid rescued Wnt receptors at both protein and mRNA levels (Fig. 1h, i and Supplementary Fig. S1b), suggesting EpCAM might have a regulatory role in Wnt receptor expression. Of note, EpCAM has been reported to act via EpEX and EpICD to influence critical cell signaling pathways that promote tumor progression. Thus, we previously developed a humanized EpCAM neutralizing monoclonal antibody, hEpAb2-6, which could inhibit EpCAM regulated intramembrane proteolysis (RIP) [9]. The antibody binds to and inhibits the cleavage of EpEX, which impedes shedding of EpICD. This action subsequently blocks EpCAM-mediated signaling and induces apoptosis in the cancer cells. The hEpAb2-6 antibody differs from other anti-EpCAM antibodies that are in clinical trials due to its unique binding sites at positions L94, Y95 and D96 in the EGF-II/TY domain of EpCAM; this binding profile allows the antibody to inhibit cleavage of both domains [9]. Interestingly, treating cells with hEpAb2-6 also significantly downregulated Wnt receptor protein and mRNA levels in multiple CRC cell lines (Fig. 1j, k and Supplementary Fig. S1c). Therefore, we next wanted to test whether inhibition of EpCAM signaling would suppress Wnt receptor expression in patient-derived samples. Notably, patient-derived organoids (PDOs) exhibit a variety of cancer phenotypes thus mimic several features of cancer progression that naturally occur in patients. Such features include relying on high levels of Wnt activity, harboring CSCs, attaining clonal dynamics and plasticity and recapitulating tumor heterogeneity and architecture [23–25]. We next utilized PDOs from our previously established live biobank [21] to further examine the influence of EpCAM on Wnt receptor expression (Fig. 1l). Given CRC tissues often have high expression of EpCAM, we first tested if hEpAb2-6 would readily bind to EpCAM on the surface of cells in PDOs using immunofluorescence staining (IFS) (Fig. 1l, m). Indeed, we observed strong interactions of hEpAb2-6 with the PDOs, in contrast to that of the negligible binding of normal IgG. Furthermore, we found that treating PDOs with hEpAb2-6 significantly decreased Wnt receptor expression at both protein and mRNA levels (Fig. 1n, o and Supplementary Fig. S1d). Thus, our results confirmed that EpCAM signaling was involved in Wnt receptor protein expression in CRC and further showed that Wnt receptor expression could be attenuated by the treatment of hEpAb2-6.

### Nuclear translocation of EpICD is required for Wnt receptor protein expression

**Fig. 2:**
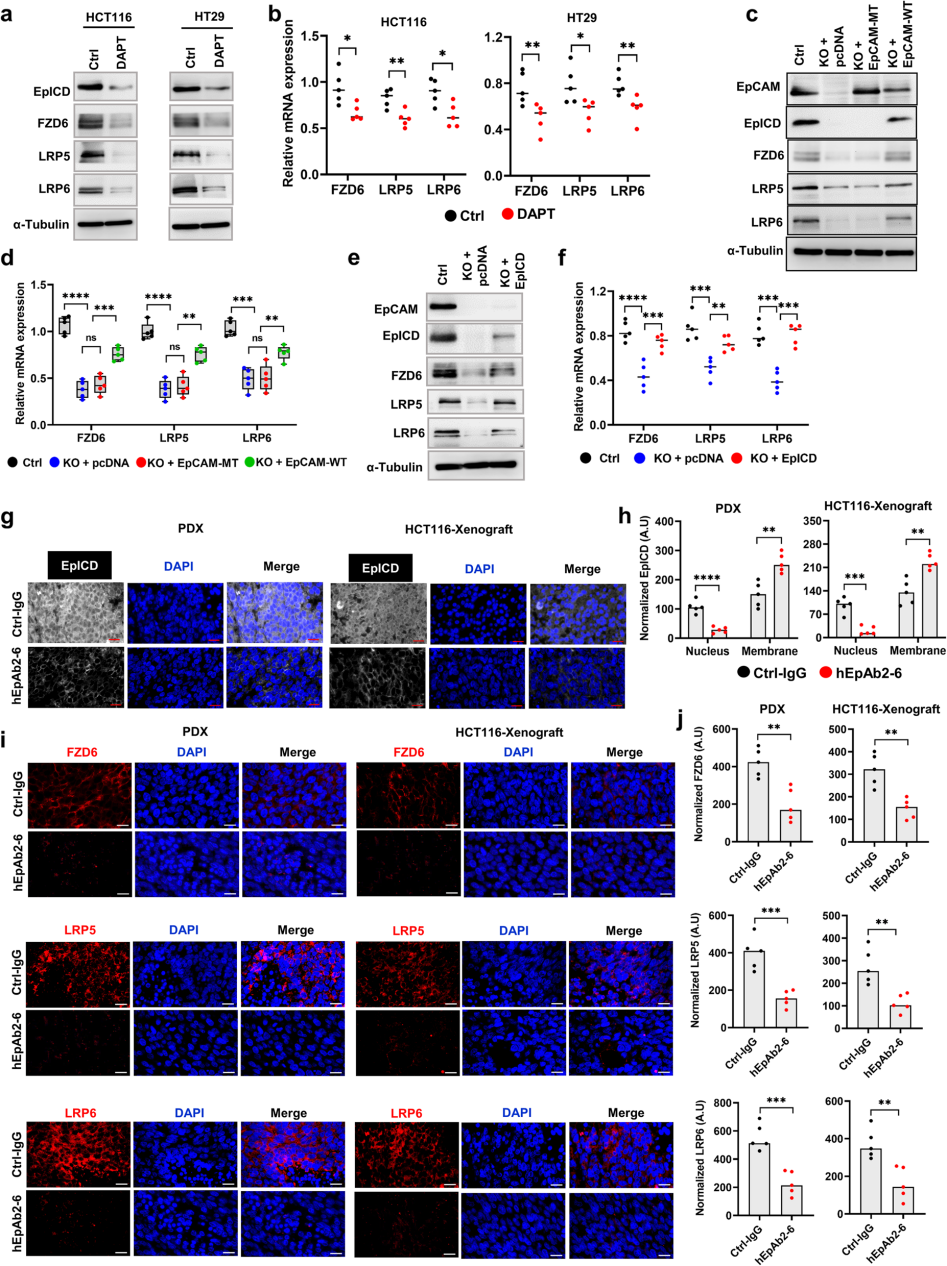
Nuclear EpICD regulates Wnt receptor expression. **a** Western blot analysis showing Wnt receptor protein expression and **b** relative mRNA expression in indicated cell lines with overnight DAPT treatment (50 μM). **c** Protein and **d** mRNA expression in control and EpCAM-KO HCT116 cells transiently transfected with either pcDNA or γ-secretase cleavage-resistant EpCAM plasmid (EpCAM-MT) or EpCAM wild-type plasmid (EpCAM-WT). The EpCAM-MT plasmid harbors EpICD cleavage site mutations, including R290E, K291E, K292E and R293E. **e** Protein and **f** mRNA expression in EpCAM-KO HT29 cells transiently transfected with either pcDNA or EpICD plasmids. Quantified band intensities from three independent experiments (**a**, **c** and **e**) are provided in Supplementary Fig. 1. **g** – **j** Either PDX (PDO#1 transplanted) or HCT116-xenograft models were generated by transplanting single cells (1 × 10^5^ cells) and allowed to grow for two weeks (~ 100 mm^3^) and then treated with either IgG or EpAb2-6 (20 mg/kg) for two weeks and then tumors were surgically isolated and processed for sectioning. Representative IFS images (scale bar: 20 μm) showing (**g**) EpICD expression and **h** quantification of membrane and nuclear EpICD staining, or (**i**) Wnt receptor expression and corresponding (**j**) quantification of staining intensity in tumors. Quantitative data were derived from *n* = 5 tumors per group; each data point is the average of two different sections from the tumor. Statistics were performed (**b**, **h**, **j**) using two-tailed t test and (**d**, **f**) one-way ANOVA followed by Tukey’s multiple comparison test. *p < 0.05, **p < 0.01, ***p < 0.001, ****p < 0.0001. Ctrl: control, KO: knockout

It is well established that many cellular effects of EpCAM depend on EpICD nuclear translocation and transcriptional promotion of EpCAM target genes [1]. Thus, we hypothesized that EpICD may act as a transcriptional co-factor for Wnt receptors. To test this hypothesis, we utilized DAPT, a small molecule inhibitor of a metalloprotease γ-secretase that was known for cleaving EpICD from intact full-length EpCAM [1, 5] (Fig. 2a, b and Supplementary Fig. S1e). As expected, DAPT treatment decreased the level of EpICD as well as the protein and mRNA expression levels of Wnt receptors. In order to further confirm the involvement of EpICD, we introduced mutations into the EpICD cleavage site within the EpCAM plasmid (EpCAM-MT), rendering the protein resistant to γ-secretase activity [26]. EpCAM-knockout cells displayed decreased Wnt receptor expression both at protein and mRNA levels. Transfecting the knockout cells with EpCAM-MT plasmid could not rescue the receptor expression but transfection of EpCAM wild-type (EpCAM-WT) plasmid did rescue the effect (Fig. 1h, i; Fig. 2c, d; Supplementary Fig. S1f). Consistent with these results, transfecting EpCAM-knockout cells with only EpICD plasmid rescued expression of Wnt receptor proteins (Fig. 2e, f and Supplementary Fig. S1g). To further study the function of EpICD in vivo, we generated patient-derived xenografts (PDX) and HCT116 cell-derived xenografts. Since hEpAb2-6-mediated inhibition of Wnt receptor expression was observed in both CRC cell lines and PDOs (Fig. 1j-o), we next treated tumor-bearing animals with hEpAb2-6 for two weeks, after which tumors were isolated, sectioned and analyzed by IFS studying Wnt receptor expression. In both PDX and HCT116-xenografts, we saw robust nuclear accumulation of EpICD in tumors from control-IgG-treated animals that however; was markedly decreased in tumors from hEpAb2-6-treated mice (Fig. 2g, h). In fact, the hEpAb2-6-treated animals harbored tumors with a remarkable increase in membrane-associated EpICD staining. These findings are consistent with the idea that the antibody treatment inhibited RIP to block shedding of EpICD and thereby suppress its nuclear translocation [5, 8, 9]. In addition, we found that hEpAb2-6 treatment significantly inhibited Wnt receptor protein levels in both xenograft models (Fig. 2i, j). Altogether, these results suggest that EpICD and its nuclear translocation are likely responsible for the upregulated expression of FZD6 and LRP5/6 observed in CRC.

### Wnt signaling activation induces γ-secretase activity in a positive feedback-loop

**Fig. 3:**
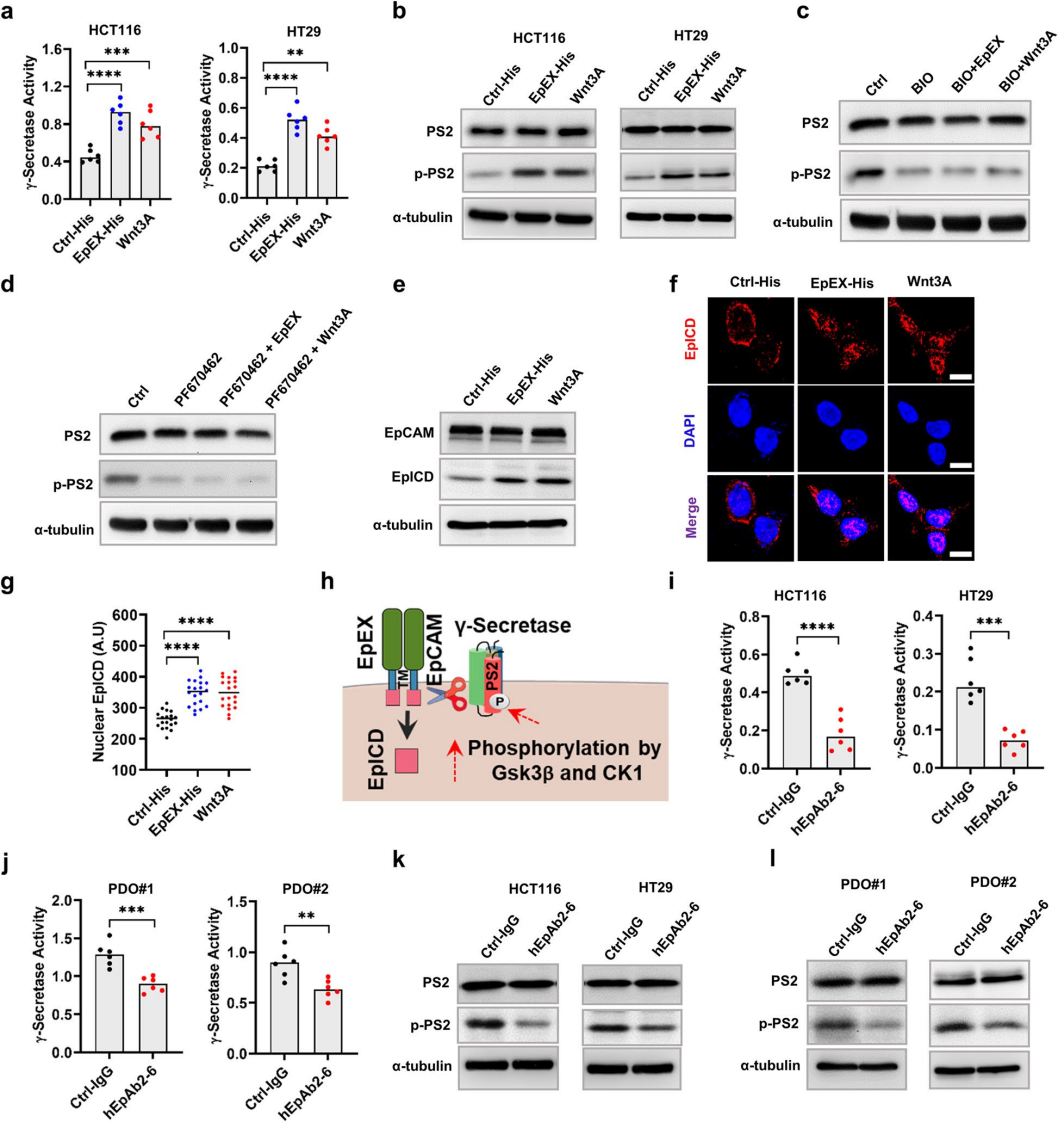
Wnt pathway activation induces γ-secretase activity stimulating EpICD production and its subsequent nuclear translocation. **a** γ-secretase activity quantified in cell lines treated with either ctrl-His or Wnt3A (100 ng/mL) or EpEX-His (250 ng/mL) for 4 h. Western blot analysis showing phosphorylated presenilin-2 (PS2) in (**b**) indicated cells treated with ctrl-His (100 ng/mL), Wnt3A (100 ng/mL) or EpEX-His (250 ng/mL) for 20 min or with 1 h pre-treated (**c**) BIO (50 µM) or (**d**) PF-670462 (50 µM) in HCT116 cells. **e** Western blot analysis showing overall EpICD level in total lysate, and **f** representative IFS images (Scale bar: 10 μm) showing EpICD localization in HCT116 cells treated with either ctrl-His or Wnt3A (100 ng/mL) or EpEX-His (250 ng/mL) for 8 h. **g** Quantification of nuclear EpICD levels from 20 different cells per group. **h** Illustration showing the proposed mechanism of PS2 phosphorylation by GSK3β and CK1 to activate γ-secretase, which then cleaves EpICD from intact full-length EpCAM. **i**-**j** γ-Secretase activity and **k**, **l** Western blot analysis showing phosphorylation of PS2 in indicated cell lines and PDOs treated with either ctrl-IgG or hEpAb2-6 (20 μg/mL) for 4 h. Data were analyzed using (**a**, **g**) one-way ANOVA followed by Tukey’s test for multiple comparisons and **i**, **j** two-tailed t test. *p < 0.05, **p < 0.01, ***p < 0.001, ****p < 0.0001. Ctrl: control

Since we found that EpICD production and its nuclear translocation were necessary for Wnt receptor (FZD6 and LRP5/6) expression, we next wanted to understand how γ-secretase was activated and how it influenced EpICD production in CRC. We thus asked whether activation of Wnt signaling influenced γ-secretase activity and modulated cleavage of EpICD. In this context, it is known that EpEX may induce EpCAM RIP and upregulate EpICD release. Thus, we tested the effects of both EpEX and a Wnt ligand (Wnt3A) on γ-secretase activity (Fig. 3a). Interestingly, we found that both treatment with exogenous EpEX or Wnt3A could significantly enhance γ-secretase activity as compared to control treatments. Since γ-secretase is typically activated via phosphorylation of its presenillin-2 (PS2) subunit, we further tested how PS2 phosphorylation was influenced by EpEX and Wnt3A treatment. We noticed both EpEX and Wnt3A treatment (Wnt signaling activation) could increase phopho-PS2 levels (Fig. 3b). In order to dissect the mechanisms of these effects, we next sought to identify the kinases involved in this process. Remarkably, blocking GSK3β or CK1 proteins of the β-Catenin destruction complex with small molecule inhibitors resulted in decreased phosphorylation of PS2 (Fig. 3c, d) suggesting activation of Wnt signaling could induce γ-secretase activity. Moreover, we also found that neither EpEX nor Wnt3A treatments could induce phosphorylation of PS2 in the presence of GSK3β and CK1 inhibitors. These results suggested that treatment with both Wnt3A and EpEX involved GSK3β and CK1 in the activation of γ-secretase (Fig. 3c, d). In order to confirm if upregulated γ-secretase activity was associated with increased EpICD cleavage, we treated the cells with exogenous EpEX and Wnt3A and measured EpICD in total cell lysates by Western blot analysis. In addition, both EpEX and Wnt3A treatment increased productions of whole-cell levels of EpICD as well as enhanced its nuclear translocation as assessed by Western blot and IFS (Fig. 3e-g). These results altogether suggested Wnt signaling activation induced γ-secretase activity by stimulating phosphorylation of PS2 with the requirements of kinases GSK3β and CK1 present in the β-Catenin destruction complex. Thus, a positive feedback loop existed in which Wnt signaling induced shedding and nuclear translocation of EpICD that further upregulated Wnt receptor expression and related Wnt signaling activity (Fig. 3h). We next tested if treatment with hEpAb2-6 could inhibit the activation of γ-secretase that might block EpICD cleavage in cell lines and PDOs. Indeed, the antibody treatment could significantly inhibit γ-secretase activity and phospho-PS2 levels compared to control IgG treatment in both cell lines and PDOs (Fig. 3i-l).

### EpICD interacts with the promoters of Wnt receptors to induce transcription

**Fig. 4:**
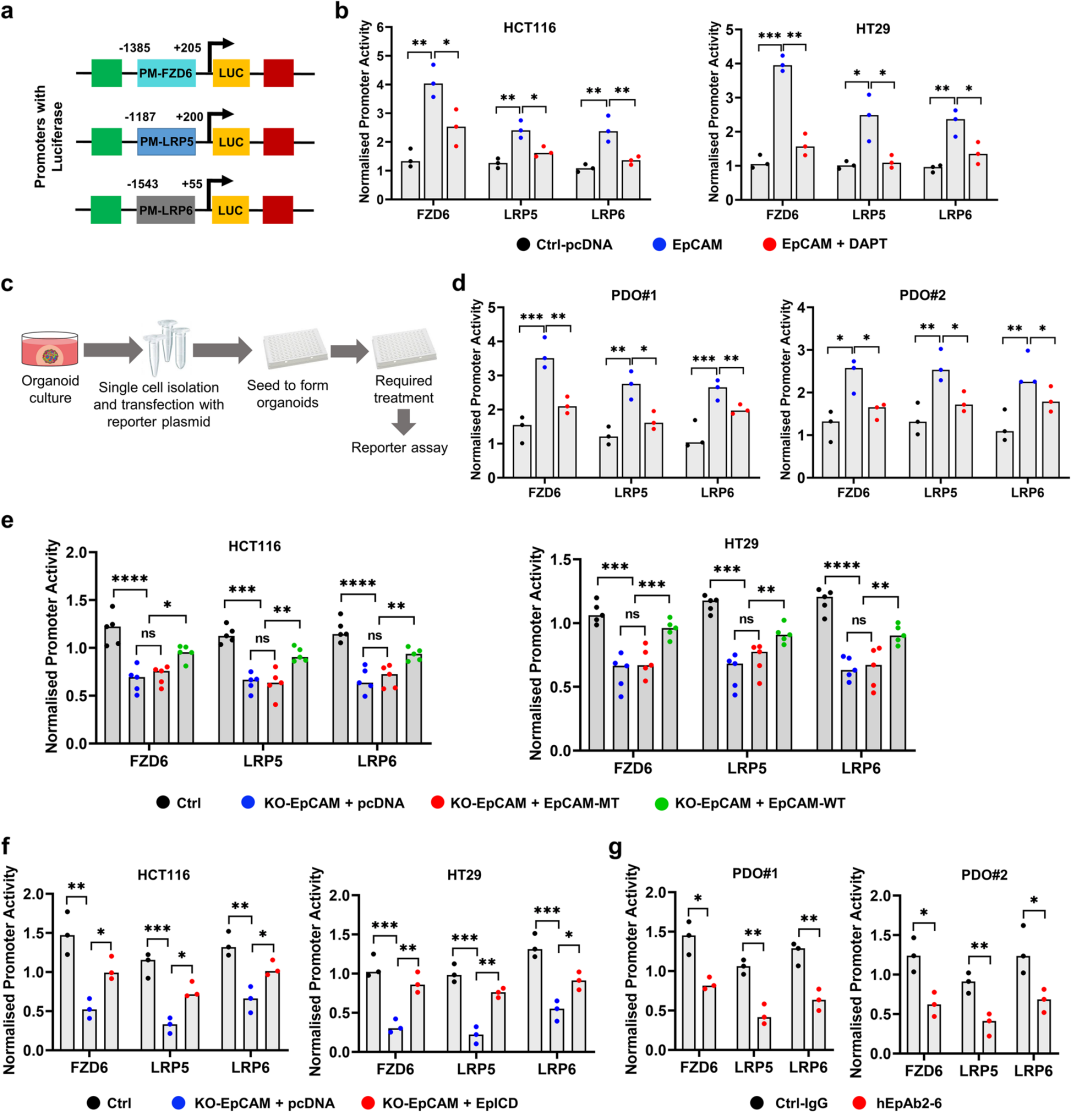
EpICD induces transcriptional activity of Wnt receptor promoters. **a** Diagram shows construction of luciferase reporters to study Wnt receptor promoter activity. **b** Indicated Wnt receptor promoter activity after transient transfection of EpCAM plasmid and overnight treatment of DAPT (50 μM). **c** Experimental procedure for the transient transfection of PDOs with reporter plasmids. **d** Promoter activity in indicated PDOs treated as in (**b**). **e** Wnt receptor promoter activity in indicated cell lines transiently transfected with pcDNA, γ-secretase cleavage-resistant EpCAM plasmid (EpCAM-MT; harbors EpICD cleavage site mutations R290E/K291E/K292E/R293E) or EpCAM wild-type (EpCAM-WT) plasmid and **f** either pcDNA or EpICD plasmid, and in (**g**) PDOs treated with either IgG or hEpAb2-6 (20 μg/mL) for 8 h. Data were analyzed using (**b**, **d**, **e** and **f**) one-way ANOVA followed by Tukey’s test for multiple comparison and **g** two-tailed t test. *p < 0.05, **p < 0.01, ***p < 0.001, ****p < 0.0001. LUC: Luciferase, KO: knockout, Ctrl: control

Up to this point, we had shown that activation of Wnt signaling induced γ-secretase activity augmenting cleavage of EpICD that translocated to the nucleus and promoted Wnt receptor expression. Therefore, we next wanted to probe the mechanisms underlying EpICD-mediated upregulation of Wnt receptor (FZD6 and LRP5/6) transcription. For this purpose, we constructed reporters in which luciferase expression was under the control of promoters of Wnt receptors (Fig. 4a). Transfecting CRC cells with wild-type EpCAM plasmid led to enhanced promoter activity that the DAPT treatment however significantly blocked the effect (Fig. 4b). Next, we developed a method to test Wnt receptor promoter activities in PDOs. We isolated single cells from matrigel-dissociated PDOs and co-transfected with reporter plasmids and then induced organoid formation using the transfected cells. The organoids were further exposed to required treatments and subjected to the reporter assay (Fig. 4c). Similar to the results from cell lines, EpCAM transfection augmented Wnt receptor promoter activity, whilst DAPT treatment blocked the effect in PDOs (Fig. 4d). In addition, EpCAM knockout CRC cells displayed reduced Wnt receptor promoter activity. The activity levels were unchanged when the cells were transfected with EpCAM-MT plasmid, but the activities were successfully rescued with EpCAM-WT plasmid (Fig. 4e). Consistently, transfecting knockout cells with EpICD plasmid also successfully restored Wnt receptor promoter activity (Fig. 4f). Finally, consistent with previous results, treatment with hEpAb2-6 significantly reduced promoter activities in PDOs (Fig. 4g). These results suggested that the nuclear translocation of EpICD and its interactions with promoters of Wnt receptors (FZD6 and LRP5/6) upregulated the promoter activities in order to induce their transcription and in turn promoted Wnt signaling.

### EpICD stimulates Wnt activity to induce CRC progression

**Fig. 5:**
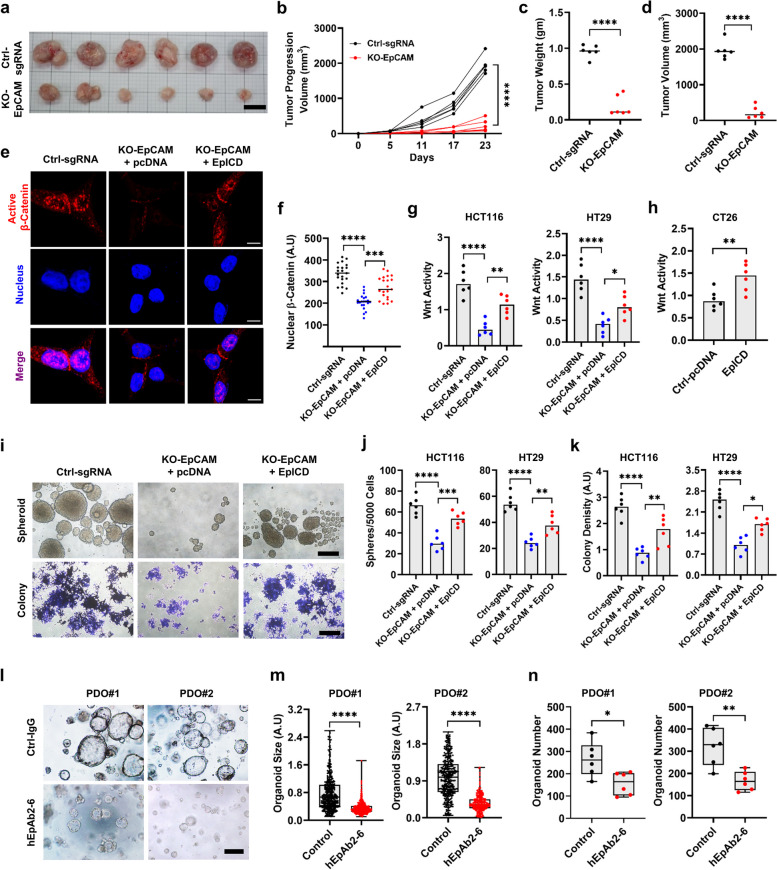
EpICD promotes Wnt-associated characteristics in CRC. **a**-**d** Comparison of individual tumor sizes and progression in vivo; 1 × 10^5^ Ctrl-sgRNA or EpCAM-KO HCT116 cells were subcutaneously transplanted in NOD/SCID mice (*n* = 6 for each cell line) (Scale bar: 1 cm). **e** Representative IFS images (Scale bar: 10 μm) showing active β-Catenin and its nuclear translocation (hallmark of canonical Wnt pathway activation) in HT29 cells transfected with either pcDNA or EpICD plasmid. **f** Quantification of nuclear β-Catenin from 20 different cells, and **g** Wnt activity as detected by TOP flash reporter assay in indicated control and EpCAM knockout cell lines with or without EpICD plasmid transfection. **h** Wnt activity in CT26 mouse colon cancer cells (do not normally express EpCAM) with either pcDNA or EpICD plasmid transfection. **i** Sphere and colony formation (Scale bar: 20 μM). **j** Sphere number and **k** colony densities in indicated cell lines (5 × 10^3^ cells seeded for both assays) transfected with either pcDNA or EpICD plasmid. **l** Images of indicated PDOs (Scale bar: 100 μm) treated with either ctrl-IgG or hEpAb2-6 (20 μg/mL) every 2 days for 6 days. Box and whisker plots showing (**m**) organoid size (from three independent wells) and **h** number (three independent experiments and two wells for each experiment) in indicated PDO cultures with indicated treatments. Data were analyzed using (**b**) two-way ANOVA, (**c**, **d**, **h**, **n**) two-tailed t test, (**f**, **g**, **j**, **k**) one-way ANOVA followed by Tukey’s test for multiple comparison and **m** Mann–Whitney U test. *p < 0.05, **p < 0.01, ***p < 0.001. Ctrl: control

We next wanted to test if EpICD-induced effects on Wnt signaling would be reflected in Wnt-associated cellular characteristics. First, we tested if EpCAM was involved in tumor formation in xenograft models (Fig. 5a). As expected, EpCAM knockout cells formed significantly smaller tumors and displayed reduced tumor progression compared to control cells (Fig. 5a-d). In addition, knocking out EpCAM significantly reduced cell growth, increasing the doubling time in HCT116 and HT29 cells (From 19.2 ± 0.7 h in controls to 40.3 ± 0.9 h in knockout HCT116 cells; likewise, from 23 ± 2 h in control to 48 ± 2 h in knockout HT29 cells) (Supplementary Fig. S2a, b). In line with these results, forced expression of EpCAM in CT26 mouse CRC cells (that normally do not express EpCAM) decreased the doubling time from 30 ± 2 h in control cells to 21 ± 2 h in EpCAM-expressing cells (Supplementary Fig. S2c). Given that EpCAM influences various stemness-related characteristics, we reasoned that EpCAM might be involved in promoting cancer cell migration and invasion. To test this idea, we performed a wound healing assay and a trans-well assay using EpCAM knockout cells. The results showed that knocking out EpCAM significantly decreased the invasion and migration properties (Supplementary Fig. S2d-g).

We thus studied if EpICD could rescue Wnt-related phenomena in EpCAM knockout cells. First, we performed IFS to examine active β-Catenin and quantify the nuclear translocation of β-Catenin (Fig. 5e, f). We noticed, EpCAM knockout significantly reduced the nuclear β-Catenin signal, and such effect could be partially rescued by transient transfection of EpICD plasmid. These results were in agreement with Wnt activity results, as measured with the TOP-Flash reporter assay (Fig. 5g). Furthermore, CT26 cells (do not express EpCAM) showed enhanced Wnt activity when transfected with EpICD plasmid (Fig. 5h). We also tested other Wnt-related properties such as spheroid and colony formation in various CRC cell lines (Fig. 5i-k). Similar to the previous results, EpICD transfection rescued these outcomes in EpCAM knockout cells. Moreover, treating PDOs with hEpAb2-6 significantly decreased PDO size and number in the cultures (Fig. 5l-n), suggesting that the EpCAM antibody suppressed Wnt-associated organoid formation. Based on our results, we concluded that EpICD-mediated transcription of Wnt receptors augmented Wnt signaling as well as drove Wnt-related phenomena in CRC.

### hEpAb2-6 suppresses tumor progression in xenograft models

**Fig. 6:**
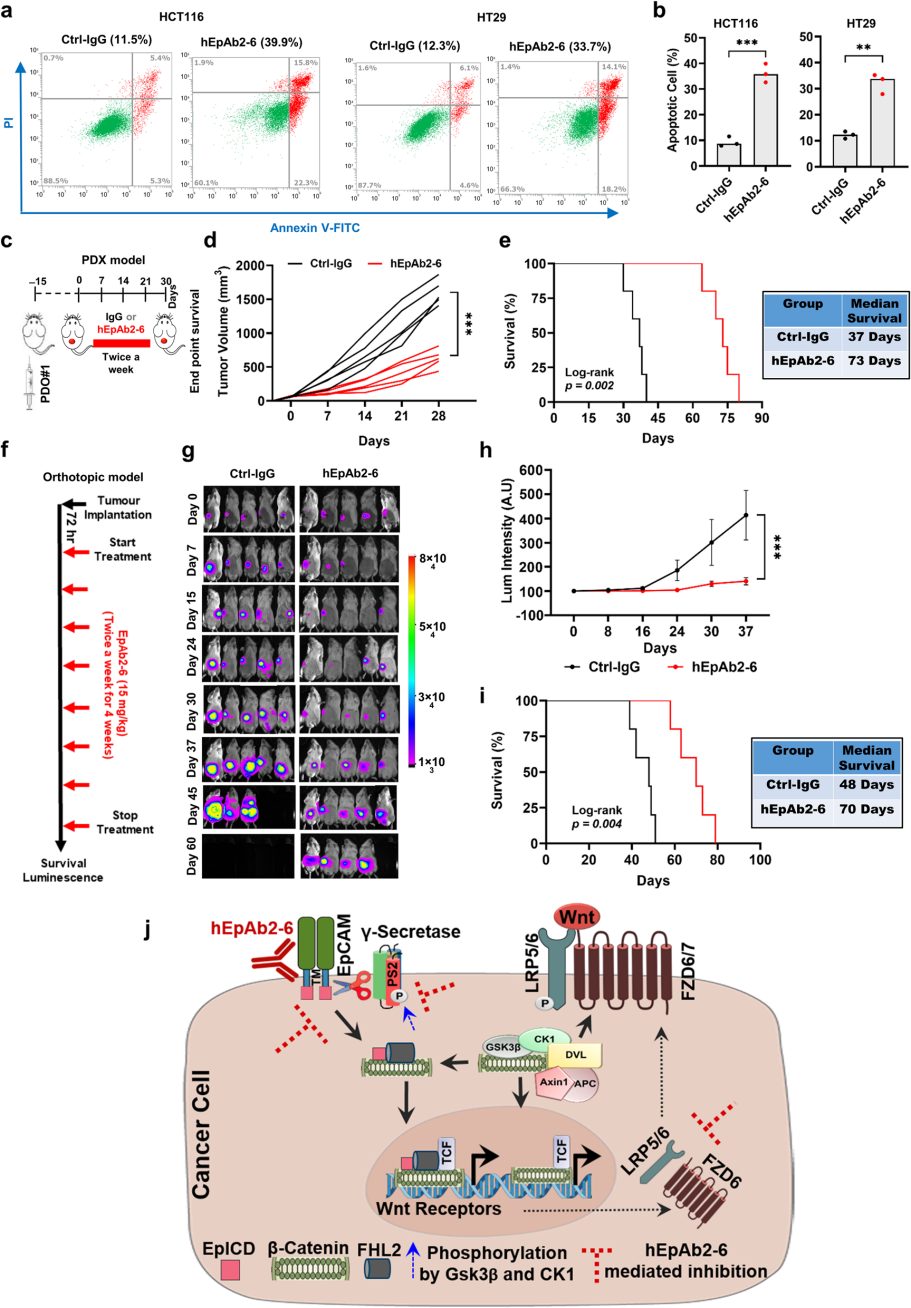
hEpAb2-6 suppresses tumor progression in multiple xenograft models of CRC. **a** Annexin V apoptosis assay after overnight treatment of hEpAb2-6 (20 μg/mL). **b** Quantification of apoptotic cell counts from three independent experiments in indicated cell lines. **c** Procedure for generating a PDX model (PDO#1) and the hEpAb2-6 treatment strategy. **d** Spaghetti plots showing individual tumor progression in animals treated bi-weekly with either Ctrl-IgG or hEpAb2-6 (20 mg/kg) for 4 weeks. The endpoint was reached when tumor volume reached 2000 mm^3^ in any mouse. **e** Kaplan–Meier survival plot showing animal survival for the PDX model (median survival displayed in adjacent table). **f** Treatment schedule of the HCT116 orthotopic animal model and **g** Bioluminescence indicating tumor progression (Day 0 = 72 h post-transplantation). **h** Quantification of luminescence intensity (mean ± standard deviation). **i** Kaplan–Meier survival plot showing animal survival for the orthotopic model (median survival is shown in adjacent table). **j** Schematic representation of the major findings in this study. EpICD regulates Wnt receptor (FZD6 and LRP5/6) expression, and hEpAb2-6 attenuates the mechanism, suggesting its potential for therapeutic application. Data were analyzed using **b** two tailed t test or (**d**, **h**) two-way ANOVA. *p < 0.05, **p < 0.01, ***p < 0.001, ****p < 0.0001. Ctrl: control

Since our data indicated that hEpAb2-6 could prevent EpICD-mediated upregulation of Wnt receptor (FZD6 and LRP5/6) transcription and related phenomena, we next sought to test whether hEpAb2-6 conferred therapeutic benefit in laboratory models of CRC. Given suppression of Wnt signaling might lead to apoptosis of cancer cells, we first tested the apoptotic effects of hEpAb2-6 in CRC cell lines (Fig. 6a, b). The results showed that the antibody significantly induced apoptosis in the CRC cells. In order to test the therapeutic effects of the antibody in vivo, we generated a PDX model by transplanting PDOs that further were treated with control IgG or hEpAb2-6 (Fig. 6c). Treatment with hEpAb2-6 significantly reduced tumor growth and progression compared to IgG treatment (Fig. 6d). In addition, the median survivals of antibody-treated animals were greater than that of the IgG-treated animals (Fig. 6e). Next, we tested the therapeutic effects of hEpAb2-6 in an orthotopic xenograft model of human CRC. To do so, HCT116 cells were surgically transplanted into the cecum wall of the animals, and the treatments were started 72 h post-transplantation (Fig. 6f). While IgG-treated animals displayed aggressive tumor progression, the hEpAb2-6-treated group showed significantly reduced tumor progression and increased median survival (Fig. 6g-i). Of note, no obvious discrepancies were observed with the safety profile of hEpAb2-6, as the antibody treatment did not cause significant decline in the bodyweight of the animals. The control IgG treatment group displayed a gradual reduction of bodyweight, probably due to the high tumor burden in both PDX and orthotopic models (Supplementary Fig. 3a, b). These results support the idea that EpICD-induced upregulation of Wnt receptor transcription and consequent activation of Wnt signaling promotes CRC progression, which however the mechanisms can be neutralized by hEpAb2-6 treatment in order to provide therapeutic benefit (Fig. 6j).

## Discussion

The tumorigenic functions of EpCAM have been widely studied in varieties of cancers. One of the most important findings is that EpCAM is expressed on the surface of CSCs and circulating tumor cells, which makes the protein a potent target for therapeutics and diagnostics [2, 3]. Although tumor-promoting functionalities of EpCAM have been extensively reported, several studies have also revealed that EpCAM exerts tumor-suppressing properties in many cancer types. Such functionalities have been studied using samples from patients with renal carcinoma, gastric cancer, colorectal cancer, lung cancer and ovarian cancer [27]. Despite the large number of studies reporting good prognosis in patients with high EpCAM expression, the exact mechanisms by which EpCAM supresses tumor progression remain unclear. In contrast, many studies have reported pro-tumor functionalities of EpCAM in patient samples for some of the same cancer types [27]. While dual functionalities of EpCAM in the context of cancer are thought to exist, the mechanistic insights behind its pro-tumor characteristics have been well established [3]. Such mechanisms mostly involve roles of EpEX and EpICD in a variety of critical cancer cell signaling cascades, such as the EGFR, HGFR and Wnt pathways [1, 4–8]. Importantly, the involvements of EpCAM in Wnt signaling was established since EpICD translocated to the nucleus along with β-Catenin and FHL2 [1]. In the nucleus, the EpICD-β-Catenin-FHL2 complex acts on EpCAM target genes, though the specific identities of these genes are not fully known. In this study, we found that EpICD nuclear translocation is associated with transcriptional upregulation of Wnt receptor proteins including FZD6 and LRP5/6. This upregulation stimulates Wnt signaling to further promote CRC tumorigenesis.

The Wnt pathway is critical for cancer progression in several cancer types including CRC. In general, Wnt signaling is activated when an external Wnt ligand interacts with at least one FZD and one LRP receptor; the activated receptor then recruits the β-Catenin destruction complex to the cell membrane, where it is dephosphorylated forming active β-Catenin and translocating to the nucleus [11]. Thus, overexpression of the Wnt receptor proteins may sensitize the cells to external ligands and augment the signaling activity [20]. Here we observed that knocking out EpCAM led to reduced nuclear translocation of β-Catenin, and this effect was partially rescued by EpICD transfection. These results might be attributed to two potential mechanisms. First, EpICD is involved in the transcription of Wnt receptors, so knockout of EpCAM should reduce expression of the receptors, limiting the interaction of Wnt ligands with the receptors and downregulating nuclear entry of β-Catenin [11, 20, 28]. Another possible mechanism could involve the formation of the EpICD-β-Catenin-FHL2 complex, which is known to translocate to the nucleus [1]. Despite these dual functions of EpICD, we did not observe complete rescue of nuclear β-Catenin after EpICD transfection to the knockout cells. This observation suggests that additional unknown mechanisms of EpCAM may facilitate nuclear entry of β-Catenin. Therefore, a specific strategy such as targeting EpCAM could be beneficial in cancers such as CRC or head and neck cancer where both EpCAM and Wnt signaling components were highly expressed. Indeed, in this study we showed that hEpAb2-6 that known to block EpICD release could inhibit EpICD-mediated expression of Wnt receptor proteins and functional effects of Wnt signaling including tumorigenesis. Ectopic EpICD may also be instrumental in cancer cell signaling processes, and the antibody may not directly block the cleavage of ectopic EpICD. Nonetheless, ectopic EpICD still requires γ-secretase activity for its successful shedding, and hEpAb2-6 may block EpICD cleavage by inhibiting γ-secretase activity, as observed in our experiments (Fig. 3). It is important to note that hEpAb2-6 also neutralizes EpEX activity, so the tumor-suppressive effects of the antibody observed in animal models are likely to be cumulative inhibitory effects on both EpEX- and EpICD-mediated mechanisms, not only the inhibition of EpICD-mediated Wnt receptor expression [5, 6, 8].

In CRC patients, high EpCAM expression is associated with poor outcomes, likely due to the critical involvement of EpICD in CRC cell function. Moreover, low levels of membrane EpICD staining intensity in CRC tumors are indicative of poor prognosis [16–18]. It has also been shown that low membrane EpICD staining corresponds to high nuclear accumulation of the protein thus poor prognosis in several other cancer types [13, 14, 19]. In line with these clinical observations, EpICD is thought to regulate cell cycle progression by impacting cyclin D expression, as *cyclin D* was identified as an EpCAM target gene [29]. The nuclear accumulation of EpICD is also associated with transcriptional activation of major stemness genes, such as *Sox2*, *Oct4* and *c-Myc* [4]. Notably, two major underlying characteristics that contribute to cancer progression are metastasis and CSC signaling that are conferred by epithelial-to-mesenchymal transition (EMT). Several reports have implicated that EpICD in the transcriptional control of EMT factors, establishes EpCAM as a dynamic regulator of the EMT process [4, 19, 30–33]. Therefore, the major involvement of EpCAM in various types of cancers (including CRC) is likely to involve the functions of EpICD in regulating critical cellular processes such as EMT, CSC signaling and metastasis.

EpCAM has been established as a key CSC antigen in CRC and several other cancer types, and it can also be considered a pivotal contributor to Wnt signaling due to the association of EpICD with β-Catenin [1, 2, 4, 33, 34]. Importantly, CSC signaling is highly dependent on the tumor microenvironment (TME) since factors in the TME interact with a multitude of receptors to stimulate CSC propagation. Among these factors in the TME, Wnt pathway activators are abundant and may stimulate the cells to drive tumor progression. As such, targeting TME components may be an effective approach for novel cancer therapeutics [11, 35, 36]. In the case of CRC, disrupting the actions of β-Catenin-stimulating Wnt signals in the TME may suppress cancer propagation. In particular, it may be possible to target major Wnt receptors, such as LRP5 and LRP6, to attenuate CRC progression by downregulating Wnt-related signaling mechanisms [28]. Of note, TME components and Wnt signaling are also critical for development of resistance to cancer therapeutics. Disease relapse is often attributable to Wnt signaling and CSCs that have established drug resistance via multiple independent mechanisms [37–39]. Therefore, targeting Wnt components may be beneficial for treating the primary cancer as well as overcoming drug resistance in any remaining cells [10, 37–39]. For instance, neutralizing mAbs against Wnt2 and Wnt3A were shown to exert anti-proliferation effects in gastric cancer and prostate cancer models respectively [39, 40]. Additionally, antibodies against Wnt receptors such as Vantictumab (OMP-18R5; mAb that blocks five Frizzled receptors/FZDs: FZD1, FZD2, FZD5, FZD7 and FZD8) and ipafricept (FZD8-Fc) are both under evaluation as potential therapeutic agents against advanced-stage solid tumors [41, 42]. In this study, we found that EpICD-mediated transcriptional upregulation of Wnt receptors leads to augmentation of Wnt signaling. Therefore, blocking EpICD cleavage may be an effective way to suppress Wnt signaling and its associated cellular characteristics. Indeed, our results showed that treatment with hEpAb2-6 could inhibit Wnt receptor expression and Wnt-associated cancer cell phenotypes.

In addition to the cell-intrinsic effects of EpICD, EpCAM can affect other cells via release of EpEX into the TME. For example, the interaction of EpEX with EGFR was previously reported to activate AKT and ERK1/2 pathways, promoting tumor progression in both lung cancer and CRC models. Furthermore, this action of EpEX-EGFR activates TACE and γ-secretase enzymes further inducing EpCAM signaling via shedding of EpEX and EpICD [5, 8]. Liang et al. also reported that in CRC, stimulation of the EpEX-EGFR-ERK1/2 axis leads to phosphorylation of PS2 and generates EpICD [5]. Here, we found that activation of Wnt signaling caused increased levels of phosphorylated PS2 via GSK3β and CK1, establishing a positive feedback loop with EpICD. While Wnt ligands are probably the major signals to induce transcription of target genes via intracellular β-Catenin, EpCAM also participates in the signaling via EpICD-mediated promotion of Wnt receptor transcription and subsequent effects on Wnt activity levels [11]. Most importantly, we showed that hEpAb2-6 treatment inhibited phospho-PS2 levels to block γ-secretase activity and attenuate EpICD-mediated cellular effects.

## Conclusions

Given that Wnt signaling is critical for CRC progression, this study clarifies the role of EpICD in CRC, revealing that it functions as a co-factor to transcribe Wnt receptor proteins. Furthermore, we showed that activation of Wnt signaling induces γ-secretase activity to augment the shedding of EpICD in a positive feedback loop. Treatment with the humanized EpCAM-neutralizing antibody, hEpAb2-6, inhibited EpICD-related mechanisms and decreased Wnt receptor expression. The antibody also attenuated tumor progression and extended survival in multiple CRC xenograft models. We therefore propose that anti-EpCAM therapeutics may be effective for the treatment of CRC.

### Supplementary Information

Additional file 1: Supplementary Figure S1. EpCAM regulates Wnt receptor protein expression via EpICD. Graphs represent quantification of band intensities from 3 independent experiments provided in (a) figure 1f, (b) figure 1h, (c) figure 1j, (d) figure 1n, (e) figure 2a, (f) figure 2c and (g) figure 2e. Data were analyzed using (a, b, f) one-way ANOVA followed by Tukey’s test for multiple comparison and (c, d, e) two-tailed t test. *p < 0.05, **p < 0.01, ***p < 0.001, ****p < 0.0001. Ctrl: control.

Additional file 2: Supplementary Figure S2. EpCAM regulates cancer cell growth and progression. Growth curve comparison of indicated cells with (a-b) EpCAM-KO and (c) EpCAM forced expression. (d) Representative images of a wound healing assay performed with CRISPR/Cas9-mediated EpCAM-knockout (KO-EpCAM) and control-gRNA (ctrl-gRNA) cell lines. The culture medium was supplemented with proliferation inhibitor Mitomycin-C, and (e) quantification of wound healing was performed for three independent experiments. (f) Transwell assay showing migration of HCT116 and HT29 cell lines. (g) Quantitative results of migrated cells (%) were derived from three independent experiments. Data were analyzed using (a, b, c and e) two-way ANOVA followed by Sidak test for multiple comparisons and (g) two-tailed t test. *p < 0.05, **p < 0.01, ***p < 0.001. Ctrl: control, KO: Knockout.

Additional file 3: Supplementary Figure S3. Animal body weights in indicated xenograft models treated with either ctrl-IgG or hEpAb2-6 as described in Fig. 6.

Additional file 4: Table S1. Primers for qPCR. Table S2. Primer for construction of Wnt receptor promoter.

## Data Availability

In response to reasonable requests, non-commercially available materials, experimental protocols and data may be provided to not-for-profit or academic requesters upon completion of a material transfer agreement. All requests should be made by contacting the corresponding author H.C. Wu.

## Abbreviations

EpCAM: Epithelial cell adhesion molecule
EpEX: Extracellular domain of EpCAM
EpICD: Intracellular domain of EpCAM
PDO: Patient derived organoid
PDX: Patient derived xenograft
CSC: Cancer stem cells
CRC: Colorectal cancer
FZD: Frizzled
LRP: Low-density lipoprotein receptor-related protein
EGFR: Epidermal growth factor receptor
HGFR: Hepatocyte growth factor receptor
RIP: Regulated intramembrane proteolysis
FHL2: Four and one-half LIM domains protein 2
EMT: Epithelial-to-mesenchymal transition
IFS: Immunofluorescence
TCF: T-Cell factor
GSK3: Glycogen synthase kinase 3
CK1: Casein kinase 1
IHC: Immunohistochemistry
PS2: Presenillin2
TME: Tumor microenvironment

## References

[1] Maetzel D, Denzel S, Mack B, Canis M, Went P, Benk M, Kieu C, Papior P, Baeuerle PA, Munz M, Gires O (2009). Nuclear signalling by tumour-associated antigen EpCAM. Nat Cell Biol.

[2] Gires O, Klein CA, Baeuerle PA (2009). On the abundance of EpCAM on cancer stem cells. Nat Rev Cancer.

[3] Gires O, Pan M, Schinke H, Canis M, Baeuerle PA (2020). Expression and function of epithelial cell adhesion molecule EpCAM: where are we after 40 years?. Cancer Metastasis Rev.

[4] Lin CW, Liao MY, Lin WW, Wang YP, Lu TY, Wu HC (2012). Epithelial cell adhesion molecule regulates tumor initiation and tumorigenesis via activating reprogramming factors and epithelial-mesenchymal transition gene expression in colon cancer. J Biol Chem.

[5] Liang KH, Tso HC, Hung SH, Kuan II, Lai JK, Ke FY, Chuang YT, Liu IJ, Wang YP, Chen RH, Wu HC (2018). Extracellular domain of EpCAM enhances tumor progression through EGFR signaling in colon cancer cells. Cancer Lett.

[6] Lee CC, Yu CJ, Panda SS, Chen KC, Liang KH, Huang WC, Wang YS, Ho PC, Wu HC (2023). Epithelial cell adhesion molecule (EpCAM) regulates HGFR signaling to promote colon cancer progression and metastasis. J Transl Med.

[7] Pan M, Schinke H, Luxenburger E, Kranz G, Shakhtour J, Libl D, Huang Y, Gaber A, Pavsic M, Lenarcic B (2018). EpCAM ectodomain EpEX is a ligand of EGFR that counteracts EGF-mediated epithelial-mesenchymal transition through modulation of phospho-ERK1/2 in head and neck cancers. PLoS Biol.

[8] Chen HN, Liang KH, Lai JK, Lan CH, Liao MY, Hung SH, Chuang YT, Chen KC, Tsuei WW, Wu HC (2020). EpCAM Signaling Promotes Tumor Progression and Protein Stability of PD-L1 through the EGFR Pathway. Cancer Res.

[9] Liao MY, Lai JK, Kuo MY, Lu RM, Lin CW, Cheng PC, Liang KH, Wu HC (2015). An anti-EpCAM antibody EpAb2-6 for the treatment of colon cancer. Oncotarget.

[10] Kahn M (2014). Can we safely target the WNT pathway?. Nat Rev Drug Discov.

[11] Nusse R, Clevers H (2017). Wnt/beta-Catenin Signaling, Disease, and Emerging Therapeutic Modalities. Cell.

[12] Batlle E, Clevers H (2017). Cancer stem cells revisited. Nat Med.

[13] Park SY, Bae JS, Cha EJ, Chu HH, Sohn JS, Moon WS (2016). Nuclear EpICD expression and its role in hepatocellular carcinoma. Oncol Rep.

[14] Ralhan R, He HC, So AK, Tripathi SC, Kumar M, Hasan MR, Kaur J, Kashat L, MacMillan C, Chauhan SS (2010). Nuclear and cytoplasmic accumulation of Ep-ICD is frequently detected in human epithelial cancers. PLoS ONE.

[15] Yu T, Ma Y, Wang H (2017). EpCAM Intracellular Domain Promotes Porcine Cell Reprogramming by Upregulation of Pluripotent Gene Expression via Beta-catenin Signaling. Sci Rep.

[16] Kim JH, Bae JM, Song YS, Cho NY, Lee HS, Kang GH (2016). Clinicopathologic, molecular, and prognostic implications of the loss of EPCAM expression in colorectal carcinoma. Oncotarget.

[17] Seeber A, Untergasser G, Spizzo G, Terracciano L, Lugli A, Kasal A, Kocher F, Steiner N, Mazzoleni G, Gastl G, Fong D (2016). Predominant expression of truncated EpCAM is associated with a more aggressive phenotype and predicts poor overall survival in colorectal cancer. Int J Cancer.

[18] Wang A, Ramjeesingh R, Chen CH, Hurlbut D, Hammad N, Mulligan LM, Nicol C, Feilotter HE, Davey S (2016). Reduction in membranous immunohistochemical staining for the intracellular domain of epithelial cell adhesion molecule correlates with poor patient outcome in primary colorectal adenocarcinoma. Curr Oncol.

[19] Sankpal NV, Fleming TP, Sharma PK, Wiedner HJ, Gillanders WE (2017). A double-negative feedback loop between EpCAM and ERK contributes to the regulation of epithelial-mesenchymal transition in cancer. Oncogene.

[20] MacDonald BT, He X: Frizzled and LRP5/6 receptors for Wnt/beta-catenin signaling. Cold Spring Harb Perspect Biol. 2012;4. 10.1101/cshperspect.a007880 PMC3504444 23209147

[21] Geevimaan K, Guo JY, Shen CN, Jiang JK, Fann CSJ, Hwang MJ, Shui JW, Lin HT, Wang MJ, Shih HC (2022). Patient-Derived Organoid Serves as a Platform for Personalized Chemotherapy in Advanced Colorectal Cancer Patients. Front Oncol.

[22] Liao YF, Wang BJ, Cheng HT, Kuo LH, Wolfe MS (2004). Tumor necrosis factor-alpha, interleukin-1beta, and interferon-gamma stimulate gamma-secretase-mediated cleavage of amyloid precursor protein through a JNK-dependent MAPK pathway. J Biol Chem.

[23] Tuveson D, Clevers H (2019). Cancer modeling meets human organoid technology. Science.

[24] de Sousa e Melo F, Kurtova AV, Harnoss JM, Kljavin N, Hoeck JD, Hung J, Anderson JE, Storm EE, Modrusan Z, Koeppen H (2017). A distinct role for Lgr5(+) stem cells in primary and metastatic colon cancer. Nature.

[25] Shimokawa M, Ohta Y, Nishikori S, Matano M, Takano A, Fujii M, Date S, Sugimoto S, Kanai T, Sato T (2017). Visualization and targeting of LGR5(+) human colon cancer stem cells. Nature.

[26] Tsaktanis T, Kremling H, Pavsic M, von Stackelberg R, Mack B, Fukumori A, Steiner H, Vielmuth F, Spindler V, Huang Z (2015). Cleavage and cell adhesion properties of human epithelial cell adhesion molecule (HEPCAM). J Biol Chem.

[27] Herreros-Pomares A, Aguilar-Gallardo C, Calabuig-Farinas S, Sirera R, Jantus-Lewintre E, Camps C (2018). EpCAM duality becomes this molecule in a new Dr. Jekyll and Mr. Hyde tale. Crit Rev Oncol Hematol.

[28] Chen GT, Tifrea DF, Murad R, Habowski AN, Lyou Y, Duong MR, Hosohama L, Mortazavi A, Edwards RA, Waterman ML (2022). Disruption of beta-Catenin-Dependent Wnt Signaling in Colon Cancer Cells Remodels the Microenvironment to Promote Tumor Invasion. Mol Cancer Res.

[29] Chaves-Perez A, Mack B, Maetzel D, Kremling H, Eggert C, Harreus U, Gires O (2013). EpCAM regulates cell cycle progression via control of cyclin D1 expression. Oncogene.

[30] Brown TC, Sankpal NV, Gillanders WE: Functional Implications of the Dynamic Regulation of EpCAM during Epithelial-to-Mesenchymal Transition. Biomolecules. 2021;11. 10.3390/biom11070956 PMC8301972 34209658

[31] Denzel S, Maetzel D, Mack B, Eggert C, Barr G, Gires O (2009). Initial activation of EpCAM cleavage via cell-to-cell contact. BMC Cancer.

[32] Hsu YT, Osmulski P, Wang Y, Huang YW, Liu L, Ruan J, Jin VX, Kirma NB, Gaczynska ME, Huang TH (2016). EpCAM-Regulated Transcription Exerts Influences on Nanomechanical Properties of Endometrial Cancer Cells That Promote Epithelial-to-Mesenchymal Transition. Cancer Res.

[33] Boesch M, Spizzo G, Seeber A (2018). Concise Review: Aggressive Colorectal Cancer: Role of Epithelial Cell Adhesion Molecule in Cancer Stem Cells and Epithelial-to-Mesenchymal Transition. Stem Cells Transl Med.

[34] Dalerba P, Dylla SJ, Park IK, Liu R, Wang X, Cho RW, Hoey T, Gurney A, Huang EH, Simeone DM (2007). Phenotypic characterization of human colorectal cancer stem cells. Proc Natl Acad Sci U S A.

[35] Wang Q, Shao X, Zhang Y, Zhu M, Wang FXC, Mu J, Li J, Yao H, Chen K (2023). Role of tumor microenvironment in cancer progression and therapeutic strategy. Cancer Med.

[36] Zhan T, Rindtorff N, Boutros M (2017). Wnt signaling in cancer. Oncogene.

[37] Vasan N, Baselga J, Hyman DM (2019). A view on drug resistance in cancer. Nature.

[38] Zhu GX, Gao D, Shao ZZ, Chen L, Ding WJ, Yu QF: Wnt/beta‑catenin signaling: Causes and treatment targets of drug resistance in colorectal cancer (Review). Mol Med Rep. 2021;23. 10.3892/mmr.2020.11744 PMC7723170 33300082

[39] Tan XY, Li YT, Li HH, Ma LX, Zeng CM, Zhang TT, Huang TX, Zhao XD, Fu L (2023). WNT2-SOX4 positive feedback loop promotes chemoresistance and tumorigenesis by inducing stem-cell like properties in gastric cancer. Oncogene.

[40] Nandana S, Tripathi M, Duan P, Chu CY, Mishra R, Liu C, Jin R, Yamashita H, Zayzafoon M, Bhowmick NA (2017). Bone Metastasis of Prostate Cancer Can Be Therapeutically Targeted at the TBX2-WNT Signaling Axis. Cancer Res.

[41] Gurney A, Axelrod F, Bond CJ, Cain J, Chartier C, Donigan L, Fischer M, Chaudhari A, Ji M, Kapoun AM (2012). Wnt pathway inhibition via the targeting of Frizzled receptors results in decreased growth and tumorigenicity of human tumors. Proc Natl Acad Sci U S A.

[42] Fischer MM, Cancilla B, Yeung VP, Cattaruzza F, Chartier C, Murriel CL, Cain J, Tam R, Cheng CY, Evans JW (2017). WNT antagonists exhibit unique combinatorial antitumor activity with taxanes by potentiating mitotic cell death. Sci Adv.

