# Supplementary figures and images for: Intracellular domain of epithelial cell adhesion molecule induces Wnt receptor transcription to promote colorectal cancer progression
Source: J Biomed Sci. 2024 Jul 15;31:72. doi: 10.1186/s12929-024-01057-y (PMC11247908; doi:10.1186/s12929-024-01057-y)

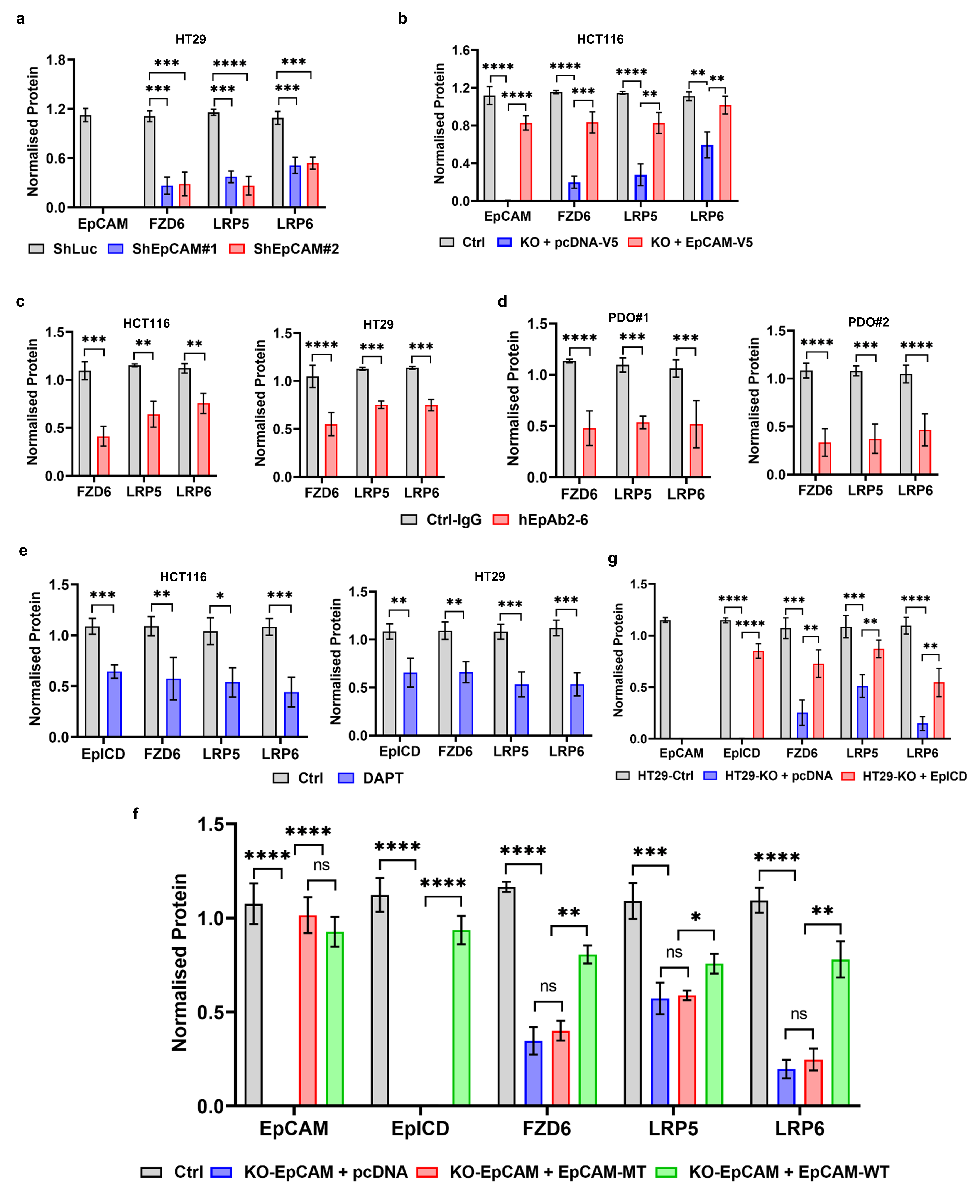

Supplement: Supplementary file 1 — Additional file 1: Supplementary Figure S1. EpCAM regulates Wnt receptor protein expression via EpICD. Graphs represent quantification of band intensities from 3 independent experiments provided in (a) figure 1f, (b) figure 1h, (c) figure 1j, (d) figure 1n, (e) figure 2a, (f) figure 2c and (g) figure 2e. Data were analyzed using (a, b, f) one-way ANOVA followed by Tukey’s test for multiple comparison and (c, d, e) two-tailed t test. *p < 0.05, **p < 0.01, ***p < 0.001, ****p < 0.0001. Ctrl: control. [file 12929_2024_1057_MOESM1_ESM.tif]

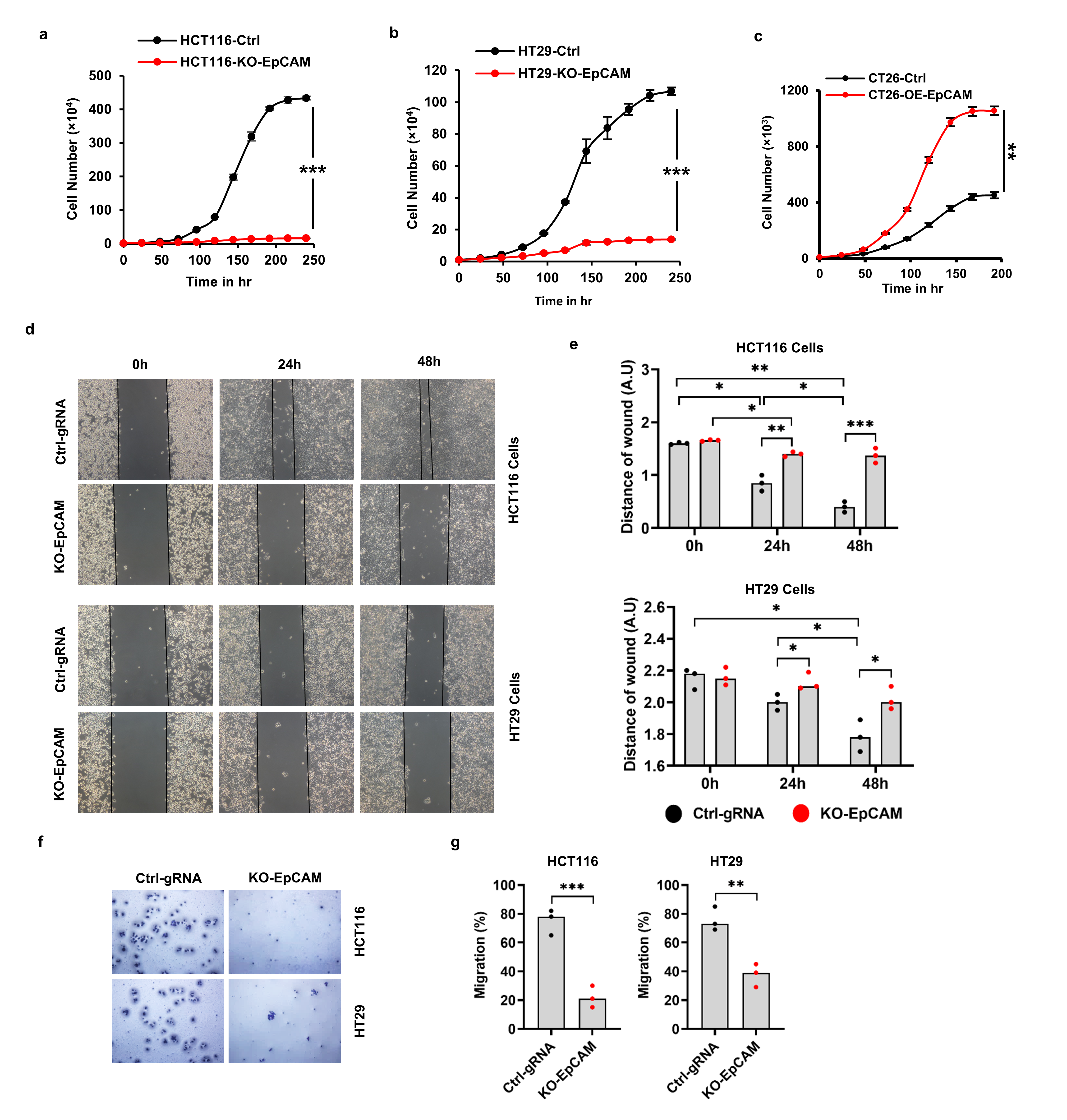

Supplement: Supplementary file 2 — Additional file 2: Supplementary Figure S2. EpCAM regulates cancer cell growth and progression. Growth curve comparison of indicated cells with (a-b) EpCAM-KO and (c) EpCAM forced expression. (d) Representative images of a wound healing assay performed with CRISPR/Cas9-mediated EpCAM-knockout (KO-EpCAM) and control-gRNA (ctrl-gRNA) cell lines. The culture medium was supplemented with proliferation inhibitor Mitomycin-C, and (e) quantification of wound healing was performed for three independent experiments. (f) Transwell assay showing migration of HCT116 and HT29 cell lines. (g) Quantitative results of migrated cells (%) were derived from three independent experiments. Data were analyzed using (a, b, c and e) two-way ANOVA followed by Sidak test for multiple comparisons and (g) two-tailed t test. *p < 0.05, **p < 0.01, ***p < 0.001. Ctrl: control, KO: Knockout. [file 12929_2024_1057_MOESM2_ESM.tif]

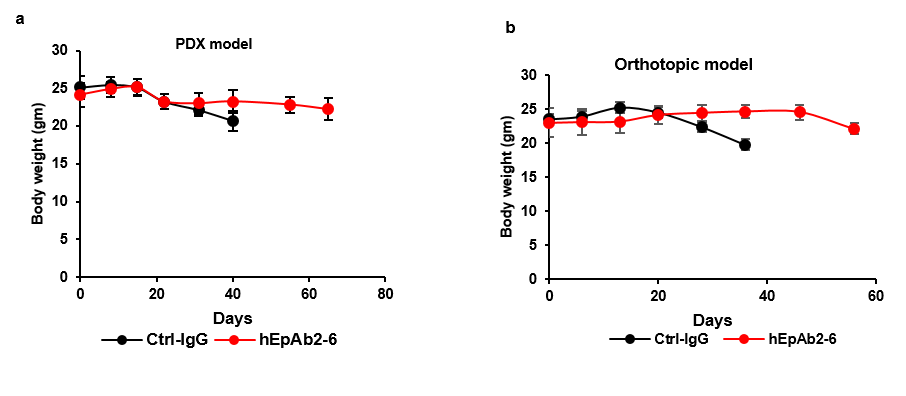

Supplement: Supplementary file 3 — Additional file 3: Supplementary Figure S3. Animal body weights in indicated xenograft models treated with either ctrl-IgG or hEpAb2-6 as described in Fig. 6. [file 12929_2024_1057_MOESM3_ESM.tif]
