# Supplementary material for: Intracellular domain of epithelial cell adhesion molecule induces Wnt receptor transcription to promote colorectal cancer progression
Source: J Biomed Sci. 2024 Jul 15;31:72. doi: 10.1186/s12929-024-01057-y (PMC11247908; doi:10.1186/s12929-024-01057-y)
Supplement: Supplementary file 4 — Additional file 4: Table S1. Primers for qPCR. Table S2. Primer for construction of Wnt receptor promoter. [file 12929_2024_1057_MOESM4_ESM.pdf]

Table S1: Primers for qPCR

Gene	Forward, Reverse
Human EpCAM	GCCAGTGTACTTCAGTTGGTGC, CCCTTCAGGTTTTGCTCTTCTCC
Human FZD6	ATTTTGGTGTCCAAGGCATC, TATTGCAGGCTGTGCTATCG
Human FZD7	GTGCAGTGTTCTCCCGAACT, GAACGGTAAAGAGCGTCGAG
Human LRP5	ACCGGAACCACGTCACAG, GGGTGGATAGGGGTCTGAGT
Human LRP6	AGGCACTTACTTCCCTGCAA, GGGCACAGGTTCTGAATCAT
Human GAPDH	AGGTCGGAGTCAACGGATTT, TAGTTGAGGTCAATGAAGGG

Table S2: Primer for construction of Wnt receptor promoter

Assay	Gene	Primer
Cloning	LRP5 PM	F: GCC GGT ACC AAG AAG GGT GGA ACC GTG TC<br>R: GCC AAG CTT TGT GGA GGG GGA TAG GGA CTT
	LRP6 PM	F: GCC GGT ACC CAG AGA CCT GGA TTG GGC TG<br>R: GCC CTC GAG TCA GGA GCA CAC AGA AGC TG
	FZD6 PM	F: CTC AGC TAG CAC CAC TGT CCC CTA<br>R: AAC ACC CTC GAG GGT GAA CGG GCT
	FZD7 PM	F: GCC GGT ACC CTA ACG CGA CTC CTG GTC AC<br>R: GCC AAG CTT TTC TCT CCG TGG TAC GGC T

PM: Promoter
